# *Sargassum fusiforme* Alginate Relieves Hyperglycemia and Modulates Intestinal Microbiota and Metabolites in Type 2 Diabetic Mice

**DOI:** 10.3390/nu13082887

**Published:** 2021-08-22

**Authors:** Jian Liu, Siya Wu, Yang Cheng, Qiuhui Liu, Laijin Su, Yue Yang, Xu Zhang, Mingjiang Wu, Jong-il Choi, Haibin Tong

**Affiliations:** 1 College of Life and Environmental Science, Wenzhou University, Wenzhou 325035, China; liujian4861@gmail.com (J.L.); wusiya1994@163.com (S.W.); ycheng19940306@163.com (Y.C.); sulaijin@126.com (L.S.); yangyue2018@wzu.edu.cn (Y.Y.); zhangx24@wzu.edu.cn (X.Z.); wmj@wzu.edu.cn (M.W.); 2 Department of Biotechnology and Bioengineering, Chonnam National University, Gwangju 500-757, Korea; 3 Bestchrom (Shanghai) Biosciences Co., Ltd., Shanghai 200120, China; qiuhuiliu@bestchrom.com

**Keywords:** *Sargassum fusiforme*, alginate, type 2 diabetes, gut microbiota, metabolome

## Abstract

*Sargassum fusiforme* alginate (SF-Alg) possess many pharmacological activities, including hypoglycemic and hypolipidemic. However, the hypoglycemic mechanisms of SF-Alg remain unclear due to its low bioavailability. In this study, we evaluated the therapeutic effect of SF-Alg on high-fat diet (HFD)/streptozotocin (STZ)-induced type 2 diabetes (T2D) mice. SF-Alg intervention was found to significantly reduce fasting blood glucose (FBG), triglycerides (TG), and total cholesterol (TC), while increasing high-density lipoprotein cholesterol (HDL-c) and improving glucose tolerance. In addition, administrating SF-Alg to diabetic mice moderately attenuated pathological changes in adipose, hepatic, and heart tissues as well as skeletal muscle, and diminished oxidative stress. To probe the underlying mechanisms, we further analyzed the gut microbiota using 16S rRNA amplicon sequencing, as well as metabolites by non-targeted metabolomics. Here, SF-Alg significantly increased some benign bacteria (*Lactobacillus*, *Bacteroides*, *Akkermansia Alloprevotella*, *Weissella* and *Enterorhabdus*), and significantly decreased harmful bacteria (*Turicibacter* and *Helicobacter*). Meanwhile, SF-Alg dramatically decreased branched-chain amino acids (BCAAs) and aromatic amino acids (AAAs) in the colon of T2D mice, suggesting a positive benefit of SF-Alg as an adjvant agent for T2D.

## 1. Introduction

Type 2 diabetes (T2D) is a global health issue in the 21st century [1]. In the later stage of T2D, a series of complications such as cardiomyopathy, hepatocellular carcinoma, and retinopathy are usually manifested, seriously affecting the patients’ health [2]. The prevalence of T2D is due to many factors, including obesity, lack of exercise, high-energy diets, as well as genetic elements [3]. Accumulated studies show that gut microbiota might participate in metabolism, immunity, and inflammation, as well as being closely related to diabetes [4]. Studies both in animals and human have revealed the differences in gut microbial composition between healthy individuals and those with T2D [5]. It has been found that the ratio of Firmicutes/Bacteroidetes in T2D individuals is higher than that in healthy individuals [6]. Furthermore, impaired intestinal permeability might be another contributing factor to the development of T2D [7]. Gut bacteria and their endotoxins can impair the intestinal barrier, resulting in the invasion of bacterial toxins or even live bacteria that would finally find their ways to the various organs, fueling the development of T2D [8]. As a bacterium that resides in mucus layer, *Akkermansia* is able to alleviate metabolic disorders induced by nutritional overconsumption, through strengthening the intestinal barrier, reducing LPS release, and controlling inflammation [9]. Attenuation of hyperglycemia by remodeling gut microbiota has also been demonstrated via oral administration of *Lactobacillus acidophilus*, or *Lactobacillus paracasei* HII01, which can lead to improved intestinal barrier function [10,11]. 

The impact of gut microbiota on T2D might be mediated through changes in the metabolites within the gut. The elevations of branched-chain amino acids (BCAAs) and aromatic amino acids (AAAs) have been found to associate with the likelihood to develop T2D [12]. Growing evidence suggests that elevated BCAAs levels may interfere with the regulation of glucose homeostasis through the excessive oxidation of the BCAAs in the skeletal muscle, resulting in abnormally high blood glucose levels [13]. In addition, the BCAAs supplement may directly promote insulin resistance and the onset of T2D in both animals and humans, possibly via the disruption of insulin signaling [14]. Gut microbiota such as Bacteroides can degrade BCAAs and AAAs, and accumulated evidence has shown that increasing the abundance of Bacteroides in the gut can lead to significant reduction of BCAAs and AAAs levels in the gut, and hence less absorption of these amino acids into the blood, thereby alleviating the symptoms of diabetes in T2D patients and animals [10,12].

Currently, there are many treatments for T2D, but there are still problems such as insufficient efficacy and good response, such as sulphonylurea and insulin, which are highly associated with increased risks of cardiovascular disease, hypoglycemia, and diabetic ketoacidosis [15,16]. Therefore, screening for novel antidiabetic leading compounds with low-toxicity and low-side effects has become an area of great interest among many researchers. As one of the remarkable modalities of complementary medicine, marine algae have created a potential platform for leading compound and drug discovery and development, especially for T2D and other metabolic diseases. Marine algae contain a variety of bioactive compounds and they present a tremendous opportunity for the discovery of new antidiabetic drug leads. Accumulating evidence suggests that bioactive compounds, such as fucoidan [17], alginate [18], and crude extracts [19] prepared from marine algae do exhibit some efficacy in the treatment of metabolic diseases such as T2D and obesity. 

*Sargassum fusiforme* is a brown alga widely distributed in the East Asian Seas. It is not only a favorite food, but also used as traditional Chinese medicine [20]. Alginate is a linear copolymer made of α-d-mannuronic acid (M) and β-l-guluronic acid (G) [21]. It has been extensively investigated that alginate is an important bioactive component and the major structural polysaccharide of *S. fusiforme* with widely biomedical applications as well as functional food ingredient [19,22]. Recently, reports on the use of alginate in the treatment of diabetes and obesity have aroused great interest among researchers. For example, alginates can lower the serum lipid level and inhibit weight gain [23]. Alginate also inhibits postprandial glucose elevation in mice by inhibiting α-amylase and α-glucosidase [18]. Although at present, a variety of alginates have been confirmed to be biologically active against inflammation, hypoglycemia, and hypolipidemia; the effects of *S. fusiforme* alginate on T2D has not been well characterized. Moreover, alginate, as biological macromolecules, is indigestible by the digestive system of host and fermentative by the gut microbiota. Further, it remains unclear whether alginate from *S. fusiforme* can alleviate T2D through remodeling gut microbiota. Therefore, this study investigated the therapeutic effects of *S. fusiforme* alginate on the hypoglycemic and hypolipidemic of diabetic mice as well as the modulation of gut microbiota and metabolites.

## 2. Materials and Methods

### 2.1. Materials

*Sargassum fusiforme* used in this study was collected from Dongtou District (Wenzhou, Zhejiang, China). Hematoxylin and eosin (H&E) staining kit was purchased from Beyotime (Shanghai, China). Streptozotocin (STZ) was purchased from Sigma-Aldrich (St. Louis, MO, USA). Blood glucose assay kit was obtained from Abbott Diabetes Care Ltd. (Chicago, IL, USA). All other chemical reagents were analytical grade. 

### 2.2. Extraction of SF-Alg

SF-Alg was extracted as previous report with some modifications [24]. Briefly, *Sargassum fusiforme* (500 g) was dried at 60 °C, and then ground and defatted with ethanol. The algal powder was further extracted with distilled water at 80 °C. After filtration and concentration, 4 M CaCl_2_ was added to the concentrate with stirring, and then the mixture was centrifuged to precipitate the calcium alginate. The above precipitate was washed once with distilled water and then collected by centrifugation to obtain calcium alginate. Next, 0.5 M HCl was add to react with the calcium alginate, and then the reaction mixture was subsequently solubilized with 4% Na_2_CO_3_ to form water-soluble sodium alginate. After dialysis and centrifugation, the solution was precipitated with ethanol to obtain sodium alginate. The precipitated sodium alginate was freeze-dried and stored under vacuum at room temperature. The final product was designated as SF-Alg (*S. fusiforme* alginate). 

### 2.3. Physicochemical Characterization of SF-Alg

The contents of uronic acid and protein were measured by m-hydroxydiphenyl method [25] and Bradford method [26], respectively. Fourier transform-infrared (FT-IR) spectroscopy analyses were conducted on a BRUKER Tensor 27 FT-IR spectrometer to characterize the *SF-Alg*. Molecular weight was determined by HPGPC [27]. 

### 2.4. Animal Experimental Design

Six-week-old male ICR mice were provided by laboratory animal center of Wenzhou Medical University. The animals were kept in the following conditions: a 12-h light and dark cycle at 22 ± 2 °C, a 60 ± 5% humidity and provided with food and water ad libitum. 

The establishment of HFD/STZ-induced T2D model was based on the previous method [28]. Following 1 week acclimatization, the mice were divided into two groups: one group was fed a normal chow diet (containing 100% AIN-93 diet), whereas the other group was fed a high fat diet (HFD, composed of 68.5% AIN-93 diet, 20% lard oil, 1% cholesterol, 0.5% sodium cholate, and 10% egg yolk powder). After 8 weeks, the HFD-fed mice were fasted for overnight and then intraperitoneally injected a single dose (40 mg/kg body weight, BW) of freshly prepared solution of STZ. After 1 week of STZ injection, fasting blood glucose (FBG) of all mice was determined. These mice with FBG levels between 11.1 mM and 33.3 mM were considered to be T2D mice. 

T2D model mice were divided into two groups, one group was administered normal saline (diabetes control, DC group), while the other group was administered SF-Alg at a dose of 100 mg/kg BW (diabetes + SF-Alg, SF-Alg group). In addition, the healthy group was also given a dose of normal saline (normal control, NC group). All treatment were administered to the animals once per day via intragastric gavage and over a period of four weeks. During the treatment period, FBG, BW, food intake, and water consumption were measured weekly.

### 2.5. Glucose Tolerance Test

The mice were fasted for 12 h and then intraperitoneally administered glucose (2 g/kg BW). Blood was taken from the tip of tail at 0, 30 min, 60 min, 90 min, and 120 min after the administration of glucose and the levels of blood glucose were measured. Changes in blood glucose levels were plotted against time to calculate the area under the curve (AUC) for IPGTT.

### 2.6. Sample Collection 

Feces were collected from the mice for gut microbiota analysis. Blood samples, collected from the mice orbit after overnight fasting, were centrifuged to isolate the serum, and then stored at −80 °C for further assay. The mice were finally euthanized via diethyl ether-induced anesthesia, and the colon, liver, heart, epididymis fat and skeletal muscle tissues were harvested from each animal. The liver, heart, epididymis fat, and skeletal muscle tissues were fixed with 10% formaldehyde. The content from the distal colon was collected for metabolomics analysis.

### 2.7. Biochemical Analysis 

Triglycerides (TG), total cholesterol (TC), low-density lipoprotein cholesterol (LDL-c), high-density lipoprotein cholesterol (HDL-c), as well as content of maleic dialdehyde (MDA), the activities of superoxide dismutase (SOD) and catalase (CAT) in the serum or liver were determined by commercial kits from Jiancheng Bioengineering Institute (Nanjing, China). Lipopolysaccharide (LPS) was determined by a ELISA kit (Jianchen Bioengineering Institute, Nanjing, China). 

### 2.8. Hematoxylin-Eosin (H&E) Staining

The epididymal adipose, liver, skeletal muscle, and heart were fixed in 10% formaldehyde, dehydrated, and then embedded in paraffin. Each specimen was sectioned at 4 μm, and stained with H&E staining kit. 

### 2.9. Gut Microbiota Analysis by 16S rRNA Amplicon Sequencing 

Then, 16S rRNA amplicon sequencing was carried out for gut microbiota analysis, which is described in full detail in the Supplementary Materials. Spearman’s correlation coefficient between gut microbiota and pathological paraments (FBG, TG, TC, LDL-c, HDL-c, serum LPS, SOD, CAT and MDA) were first calculated by the corr.test function of the psych package in R (V2.15.3). Then, the significance of the results was evaluated and visualized by the pheatmap function in the heatmap package.

### 2.10. Non-Targeted Metabolome Analysis 

Non-targeted metabolome analysis of the contents of distal colon was performed with a LC-MS method using Waters ACQUITY UPLC (Waters, Milford, MA, USA) coupled to a Thermo LTQ Orbitrap XL system (Thermo, Waltham, MA, USA), and the details of the method were described in the Supplementary Material.

### 2.11. Statistical Analysis

Statistical analysis of the data was performed using one-way ANOVA and Duncan’s multiple comparison test with the SPSS software (version 21; SPSS). Statistical significance was considered at the *p* < 0.05 level.

## 3. Results 

### 3.1. Physicochemical Properties of SF-Alg

The yield of SF-Alg was 25.2%, and it contained 92.65% uronic acid and no protein was detected. The average molecular weight of SF-Alg was calculated to be 224.651 kDa. In the FT-IR spectrum as depicted in Figure S1, two absorption bands around 3426 cm^−1^ and 2930 cm^−1^ corresponding to stretching of O–H and C–H stretching vibrations, respectively. The absorption peak at 1615 cm^−1^ may be attributed to carboxylate O–CO– asymmetric stretching vibrations. The symmetric stretching vibrations peaks around 1419 cm^−1^ may be assigned to C–OH deformation vibrations with contribution of O–CO– of carboxylate group. Additionally, the two weak bands, at about 872 cm^−1^ and 817 cm^−1^ were associated with the l-guluronicacid asymmetric ring vibration and to the d-mannuronic acid, respectively.

### 3.2. SF-Alg Alleviates Disturbance in Glucose Metabolism and Serum Lipid

**Figure 1:**
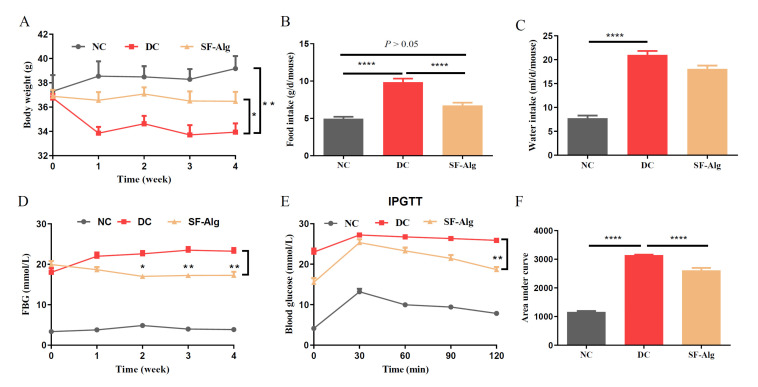
SF-Alg alleviates symptoms in HFD/STZ-induced diabetic mice. (**A**) Body weight, (**B**) food intake, (**C**) water intake, (**D**) FBG, (**E**) IPGTT, and (**F**) area under curve of IPGTT are shown. Data are presented as mean ± SEM (*n* = 8). ‘*’, ‘**’ and ‘****’ indicate significantly different from the DC group at the *p* < 0.05, *p* < 0.01 and *p* < 0.0001 levels, respectively.

It is a classical method to establish mouse model of T2D induced by HFD feeding and STZ injection. HFD/STZ-induced diabetic mice usually show gradual emaciation, decreased BW, and increased water consumption and food intake compared with the normal mice [12,29]. The BW of the diabetic mice (DC group) was no higher than that of the healthy mice (NC group) during the initial week (0 week) of SF-Alg intervention, since STZ injection cuased a dramtically loss of BW. There was a gradual downward trend in the BW of DC group during the four weeks of the experiment. But when the diabetic mice were administered SF-Alg (SF-Alg group), a significant (*p* < 0.05) reduction in weight loss and food intake was observed from week 1 to week 4 (Figure 1A,B), while there was no significant difference in water intake during the treatment period (Figure 1C). Compared with the DC group, the FBG level in SF-Alg group showed a significant decrease from the second week onward (Figure 1D). In addition, IPGTT results (Figure 1E) showed a more pronounced trend of blood glucose decrease in the SF-Alg group after 60 minutes of intraperitoneal glucose injection compared to the DC group, and the AUC of IPGTT (Figure 1F) further indicated that SF-Alg significantly (*p* < 0.001) improved glucose tolerance in diabetic mice.

Diabetic individuals often have dyslipidemia, causing the increased plasma TG, TC, LDL-c, and decreased HDL-c. Consistent with the results of other studies [30], we found that the diabetic mice (both DC and SF-Alg groups) also exhibited higher levels of TG, TC and LDL-c and a lower level of HDL-c compared with the healthy mice (NC group). However, diabetic mice treated with SF-Alg showed a significant (*p* < 0.05) decrease in the levels of TG and TC, and increase in HDL-c level (Table 1). Taken together, these data above showed a potential mitigation effect of SF-Alg on T2D.

### 3.3. SF-Alg Improves Pathological Morphology and Oxidative Stress in Diabetic Mice

**Figure 2:**
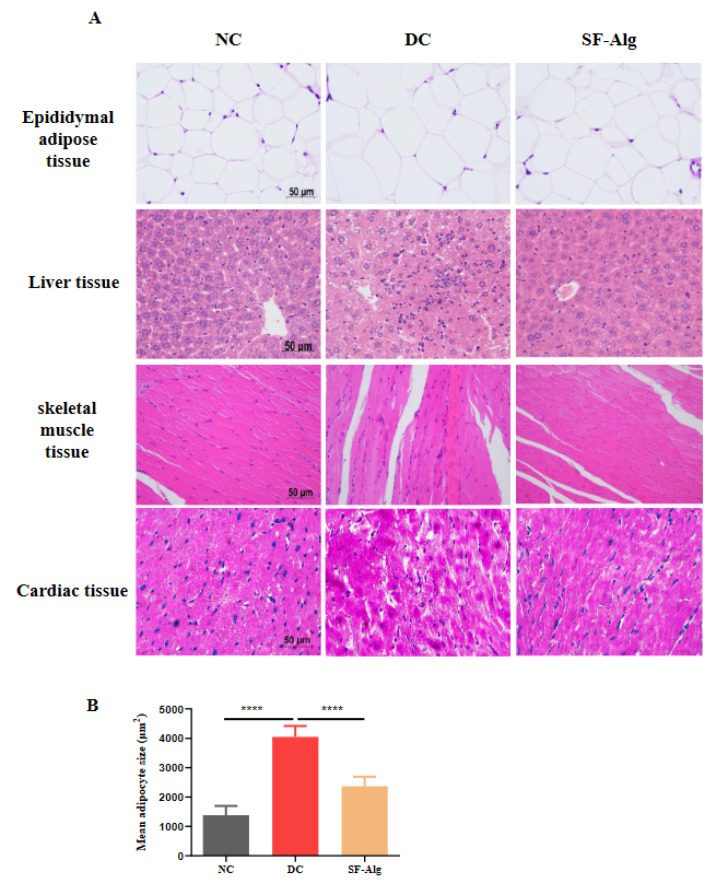
SF-Alg improves the pathological morphology of diabetes. (**A**) Epididymal adipose tissue, liver, skeletal muscle and heart samples were fixed in 10% formaldehyde, paraffin-embedded, sectioned, and stained with H&E staining kit. Images were acquired with a Leica DM3000 microscope at 400 magnifications (scalebar, 50 μm). (**B**) The average size of adipocyte was quantitatively analyzed using ImageJ software. ‘****’ indicate significantly different from the DC group at the *p* < 0.0001 level.

Diabetes causes various complications, such as diabetic cardiomyopathy, chronic liver disease, which are the main causes of mortality in the diabetic patients [31]. Therefore, the histopathological morphology of the diabetic mice was further evaluated using H&E staining. Compared with DC group, mice in the SF-Alg group exhibited a significant (*p* < 0.001) decrease in size of epididymal adipocytes (Figure 2A,B) as well as a reduction of liver inflammation as indicated by infiltrated leukocytes and swelling hepatocytes (Figure 2A). In addition, compared with DC group, mice in the SF-Alg group also displayed fewer severe histological damages both in the muscle and cardiac tissues, such as the loss of striations and focal degenerating myocytes (Figure 2A). These data indicated that SF-Alg intervention could alleviate the histopathological conditions associated with diabetes to some extent.

**Figure 3:**
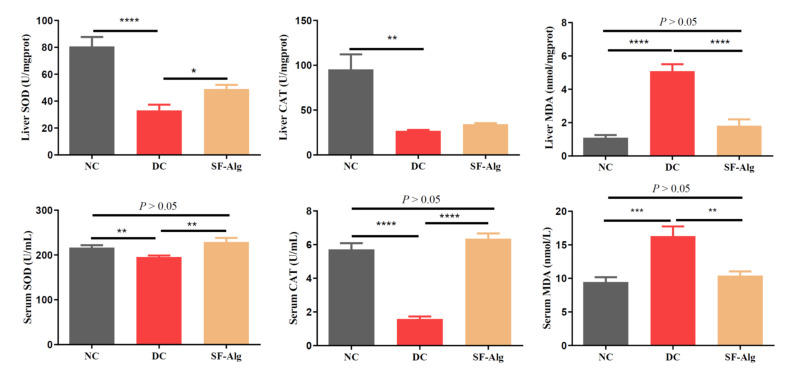
SF-Alg ameliorates oxidative stress in HFD/STZ-induced diabetic mice. The activities of SOD and CAT, and MDA level both in the liver and serum were determined using commercial kits. Data are presented as mean ± SEM (*n* = 8). ‘*’, ‘**’, ‘***’ and ‘****’ indicate significantly different from the DC group at the *p* < 0.05, *p* < 0.01, *p* < 0.001 and *p* < 0.0001 levels, respectively.

Diabetes is highly associated with the over-accumulated free radicals [32]. Thus, it was reasonable to evaluate the effect of SF-Alg on lipoperoxides, such as MDA, and antioxidant enzymes, such as CAT and SOD, in the serum and liver of HFD/STZ-induced diabetic mice. As shown in Figure 3, compared with the NC group, the levels of SOD and CAT activities in liver and serum were much lower (*p* < 0.01) in the DC group, which was consistent with the loss of antioxidant capacity in the STZ-induced diabetic mice [33]. Compared with the DC group, mice in the SF-Alg group showed a significant (*p* < 0.05) increase in SOD activity in the serum and liver, as well as a significant (*p* < 0.001) increase in CAT activity in the serum. Likewise, MDA levels in liver and serum were significantly (*p* < 0.01) lower in the SF-Alg group compared to the DC group. Overall, the data suggested that SF-Alg could diminish oxidative stress by increasing the activity of antioxidant enzymes to a certain extent. 

### 3.4. SF-Alg Remodels Gut Microbiota in Diabetic Mice

**Figure 4:**
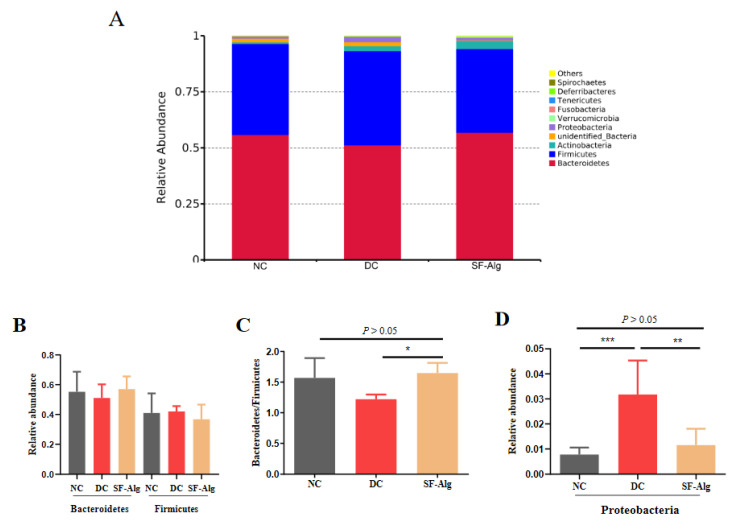
SF-Alg modulates the composition of gut microbiotain the diabetic mice at the phylumlevel. The 16S rRNA amplicon sequencing was carried out to analyze gut microbiota. (**A**) Relative abundance of gut microbiota at the phylum level, (**B**) relative abundance of Bacteroidetes and Firmicutes; (**C**) the ratio of Bacteroidetes to Firmicutes; (**D**) relative abundance of Proteobacteria. Data are presented as mean ± SEM (*n* = 8). ‘*’, ‘**’, and ‘***’ indicate significantly different from the DC group at the *p* < 0.05, *p* < 0.01, and *p* < 0.001 levels, respectively.

In order to investigate the effect of SF-Alg on the diabetic gut microbiota, 16S rRNA amplicon sequencing was carried out to detect the changes of gut microbial community. Reads were cut and filtered, and 84,652 reads were measured per sample on average. A total of 80,730 valid reads were obtained on average after quality control. The average valid reads obtained for each sample were 80,101 ± 1384.81 for the NC group, 81,598 ± 5256.35 for the DC group, and 80,491 ± 981.53 for the SF-Alg group. These data were used for subsequent analysis and were clustered into OTUs (Operational Taxonomic Units) based on 97% similarity (Table S1). SF-Alg group displayed a remarkable increase in the richness of gut microbiota (Chao1 and Ace) compared with the DC group. The clearly separated spots between groups were revealed by the Principal Co-ordinates Analysis (PCoA) (Figure S2A) with weighted UniFrac, the Non-Metric Multi-Dimensional Scaling (NMDS) (Figure S2B), suggesting that there may be a higher similarity in structure of gut microbiota between the SF-Alg and NC groups than between the SF-Alg and DC groups. In addition, the composition of gut microbiota in the SF-Alg group was studied at the phylum and genus levels. At the phylum level, the microbial community structure was dominated by Firmicutes and Bacteroidetes (Figure 4A). The DC group displayed a decrease in the abundance of Bacteroidetes and an increase in the abundance of Firmicutes compared to the NC group. There was a modest increase in Bacteroides combined with a decrease in Firmicutes in the SF-Alg group compared with the DC group (Figure 4B). Moreover, the SF-Alg group exhibited a remarkable (*p* < 0.05) increase in the ratio of Bacteroidetes to Firmicutes (Figure 4C), and significant (*p* < 0.05) decrease in the relative abundance of Proteobacteria as well as serum level of LPS (Figure 4D and Figure S3).

**Figure 5:**
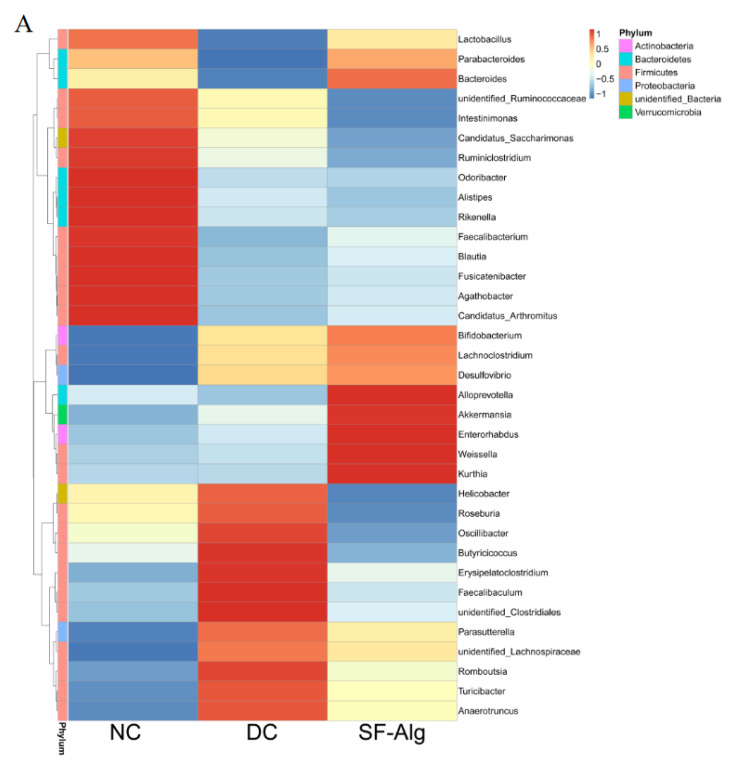

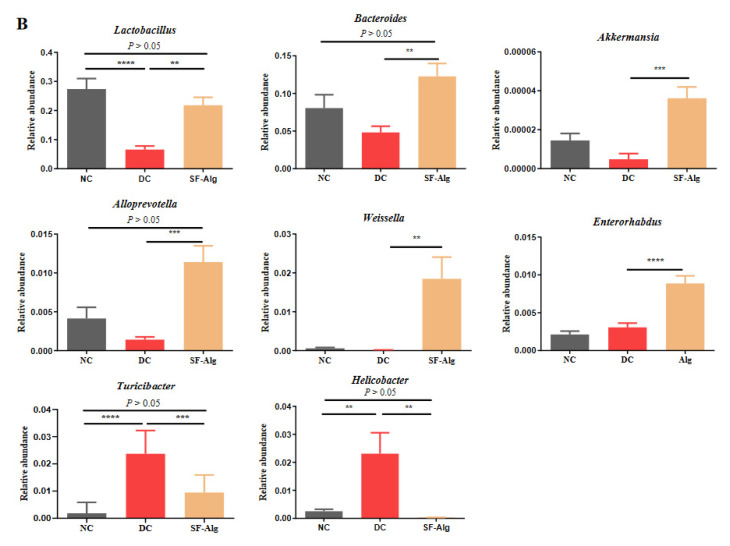
Genus-based comparison and microbial similarities. (**A**) Heatmap represents clustering of bacterial communities with their relative abundances at the genus level. Relative abundances are displayed in a red-to-blue color code (high to low abundance). (**B**) Relative abundance at genus level, including *Lactobacillus*, *Bacteroides*, *Alloprevotella*, *Akkermansia*, *Weissella*, *Enterorhabdus*, *Turicibacter*, and *Helicobater* are shown. ‘**’, ‘***’, and ‘****’ indicate significantly different from the DC group at the *p* < 0.01, *p* < 0.001, and *p* < 0.0001 levels, respectively.

**Figure 6:**
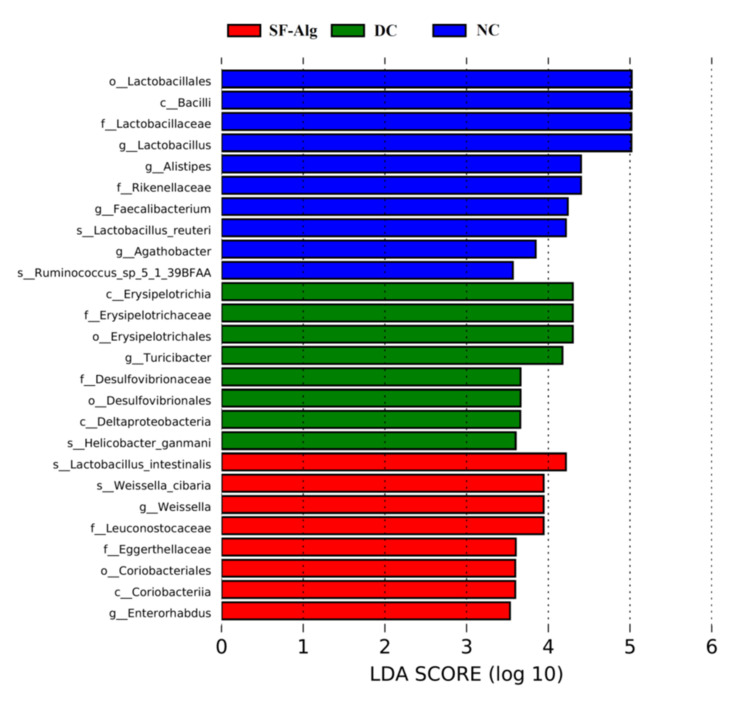
Linear discriminant analysis (LDA). Where LDA score threshold > 3.5 were listed. Blue, green, and red bars represent taxa that significantly increased in the NC, DC, and SF-Alg groups, respectively.

The relative abundance of gut microbiota at the genus level was further analyzed using the cluster as a heatmap (Figure 5A). Compared with DC group, the relative abundances of *Lactobacillus* in the SF-Alg group was significantly improved, exhibiting a similar partten to that in the NC group. Moreover, SF-Alg intervention in diabetic mice specifically boosted (*p* < 0.05) the abundance of several bacteria such as *Bacteroides*, *Akkermansia*, *Alloprevotella*, *Weissella* and *Enterorhabdus*. On the contrary, *Turicibacter* and *Helicobacter* abundance were significantly (*p* < 0.05) lower after SF-Alg supplementation in the DC group (Figure 5B). Next, LEfSe was used to identify biomarkers presented as taxons among all the three groups (Figure 6). Consistently, the mice in NC group were characterized by a higher abundance of *Lactobacillus reuteri*, *Alistipes* and *Faecalibacterium*. While, the diabetic mice in the DC group showed dramatic enrichment in *Helicobacter ganmani*, *Turicibacter*, *Desulfovibrionales*, whereas these genera are positively correlated with obesity and inflammatory disorders [34]. The relative abundance of *Lactobacillus intestinalis*, *Enterorhabdus* and *Weissella cibaria* was significantly increased in the SF-Alg group. To further study the association between changes in intestinal microbiota and a series of symptoms of diabetes, Spearman’s correlation analysis was performed between these parameters (Figure S4). *Lactobacillus* abundance was positively correlated with the levels of CAT both in serum and liver, while negatively correlated with LDL-c. *Weissella* abundance was positively correlated with HDL-c and CAT in serum and negatively correlated with MDA in serum. In addition, *Turicibacter* abundance was positively correlated with the levels of FBG, TG, TC, LDL-c, and LPS, and negatively correlated with the activities of SOD and CAT in liver. 

### 3.5. SF-Alg Modulates the Colonic Metabolome of Diabetic Mice 

**Figure 7:**
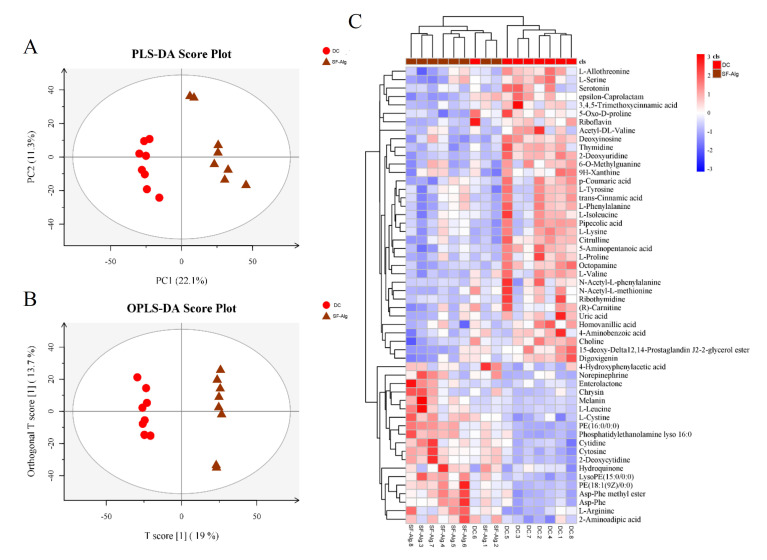
SF-Alg reshapes the metabolite profile of distal colon in diabetic mice. Discrimination of the metabolome for colon contents between SF-Alg and DC groups as indicated by PLS-DA (**A**) and OPLS-DA (**B**) score plots derived from the LC-MS data. (**C**) Heatmap indicates differential metabolites between SF-Alg and DC group at VIP > 1 and *p* < 0.05 (*t* test).

To further reveal the underlying mechanisms, metabolomics was conducted using the contents collected from the distal colons. According to the results of least squares-discriminant analysis (PLS-DA) and orthogonal partial least-squares discriminant analysis (OPLS-DA), distinctly separated clusters were observed (Figure 7A,B), exhibiting distinct metabolic profiles between untreated and SF-Alg-treated diabetic mice. In addition, 54 differential metabolites were identified according to the variable importance in the projection (VIP) > 1 and *p* < 0.05. Metabolites that displayed variation in levels between the DC and SF-Alg groups were further analyzed, and the mean normalized quantities of these metabolites were displayed as a heatmap (Figure 7C). Among the metabolites, BCAAs (l-isoleucine and l-valine) and AAAs (l-phenylalanine and l-tyrosine) displayed significantly (*p* < 0.05) reduced levels in the SF-Alg group, but their levels were increased in the DC group (Figure S5).

## 4. Discussion

In the past decade, growing evidence have demonstrated that alginate from brown algae can prevent the development of metabolic disorders. For example, calcium alginate extracted from brown seaweed can suppress HFD-induced increase in serum TG and body fat in rats [23], and polymannuronic acid (one kind of alginates isolated from brown seaweeds) can alleviate obesity and inflammation by reducing HFD-induced BW gain, fat mass, serum levels of TG and LPS, and modulating gut microbiota in mice [35]. In addition, alginate extracted from *Laminaria japonica* can also prevent a sharp rise in the level of postprandial blood glucose that might be related to its inhibition of α-amylase and maltose [18]. Therefore, the application of natural alginate has been considered as a promising strategy for the treatment of metabolic syndrome, such as obesity and T2D. Consistent with previous studies [18,23,35], we found that SF-Alg, an alginate from *S. fusiforme*, dramatically attenuated hyperglycemia and hyperlipidemia in diabetic mice. The attenuation of hyperglycemia and hyperlipidemia in these animals was accompanied by a reduction in water and food intake, weight loss, blood lipid level, as well as insulin resistance. It is well known that hyperglycemia can increase the production of free radicals and consequently oxidative stress, which is highly associated with the progression of diabetes-related complications and damages to tissues and organs [32]. SF-Alg treatment could also improve the pathological injury of the tissue and enhance the antioxidant capacity of the diabetic animals by increasing the levels of SOD and CAT activities, while decreasing the level of MDA (Figure 2 and Figure 3). Therefore, our data suggested that the ability of SF-Alg to restore part of the antioxidant defense system may be one of the important mechanisms responsible for the improvement of HFD/STZ-induced pathological symptoms in diabetic mice. 

Gut microbiota exhibits a critical role in many physiological activities, including the modulation of intestinal immune, intestinal endocrine functions, integrity and gut barrier, biosynthesis of neurotransmitters, vitamins, steroid hormones, as well as the metabolism of BCAAs, AAAs, bile salts, and drugs [36]. Accumulated evidence have indicated that gut dysbiosis is closely related to the development of T2D [8,37]. Hence, looking for potential anti-diabetic agents to balance the dysbiosis of gut microbiota might be a new therapeutic strategy for T2D. Accumulating studies have reported that the supplementation of dietary fiber can regulate intestinal microbiota. For instance, insulin can delay the development of diabetes via modulation of microbiota homeostasis [38]. In this study, SF-Alg was shown to improve the symptoms of diabetes, remodel gut microbiota, and transform the metabolites of colon content in the diabetic mice. A decline in ratio of Bacteroidetes/Firmicutes is considered as a marker of microbial imbalance, which might be linked to the development of T2D [6]. Therefore, increasing the Bacteroidetes/Firmicutes ratio in the gut might be an important factor that could interfere with the development of T2D. Multiple indigestible dietary fibers, including alginate, inulin, pectin, and arabinogalactan, can be fermented by gut microbiota [22]. Bacteroidetes is the major phylum of bacteria for the fermentation of complex carbohydrates [39]. Previous study has demonstrated that dietary alginate can elevate the abundance of Bacteroidetes [40]. Our data also showed that *S. fusiforme* alginate can increase the abundance of Bacteroidetes, while decrease the abundance of Firmicutes (Figure 4B), resulting in an increased Bacteroidetes/Firmicutes ratio (Figure 4C). In addition, studies have found that HFD-induced diabetic inflammation, oxidative stress, and metabolic disorders, are strongly correlated with endotoxemia and increased blood LPS levels [7]. Higher serum LPS can result from increased production of endotoxin upon changes in the gut microbiota [8]. It is well-known that Proteobacteria belongs to gram-negative bacteria, which the main compounds of the outer membranes are LPS [41]. Proteobacteria population was mainly enriched in the DC group, which may be responsible for the increase of serum LPS level in DC group. While, with SF-Alg treatment, the relative abundance of Proteobacteria and serum level of LPS in diabetic mice were decreased and approached that of normal mice, suggesting that SF-Alg may reduce inflammation by regulating gut microbiota. 

Moreover, at the genus level, the abundance of *Lactobacillus*, *Bacteroides*, *Akkermansia*, *Alloprevotella*, *Weissella*, and *Enterorhabdus* in the diabetic mice was also increased by SF-Alg treatment (Figure 5B). *Lactobacillus* is a typical probiotic, which is beneficial to host health, including the regulation of glucose, lipid metabolism, and the improvement of oxidative stress and inflammatory response [10,34]. In addition, our studies showed that *Lactobacillus* abundance was positively correlated with CAT, suggesting its potential role in alleviating oxidative stress. Notably, the enrichment of *Akkermansia* colonizing in the mucosal layer can reduce the severity of a diverse range of metabolic disorders, such as glucose intolerance, insulin resistance and obesity [9]. In addition, *Akkermansia* can also produce immune stimulants, enhance the gut barrier, and reduce the release of LPS. SF-Alg also reduced the relative abundance of *Helicobacter* and *Turicibacter* in diabetic mice (Figure 5B), while the *Turicibacter* abundance was positively correlated with LPS (Figure S4). In line with this, *Helicobacter* has been demonstrated to be positively correlated with insulin resistance and diabetes [42], while bacteria of the genus *Turicibacter*, considered as putatively pro-inflammatory taxa, were highly associated with diet-induced obesity [43,44,45]. Based on these findings, SF-Alg can improve the glucose metabolism as well as restore the unbalanced gut microbiota.

BCAAs and AAAs are essential amino acids that account for more than 20% of the amino acids in a typical “Western diet” [46]. They are normally utilized for the biosynthesis of protein, but once provided in excess they are diverted away from protein synthesis and toward energy utilization. Recent studies [47,48] have shown that BCAAs and AAAs are associated with disorders in energy balance, obesity, insulin resistance, and metabolic syndrome. Reductions in BCAAs and AAAs correlate with improved insulin sensitivity, as estimated by hyperinsulinemic–euglycemic clamp or homeostatic model assessment of insulin resistance (HOMA-IR) [49]. Mechanistically, BCAAs and AAAs suppress insulin-induced PI3K signaling and stimulate the activation of the mammalian target of rapamycin (mTOR), promoting the inappropriate phosphorylation of IRS-1 and further impairing insulin signaling transduction [50]. Through the analysis of differential metabolites, a significant decrease in the levels of l-valine, l-isoleucine (BCAAs) and l-tyrosine, l-phenylalanine (AAAs) were detected in SF-Alg-treated diabetic mice compared with untreated mice (Figure 7C and Figure S5). Significant negative correlation between BCAAs and *Bacteroides* species has been observed [51], consistent with our observation that SF-Alg could increase the abundance of *Bacteroides* genus in the diabetic mice. Taken together, our result suggested that SF-Alg might improve the abundance of certain bacteria, especially *Bacteroides*, so as to increase the efficiency of BCAAs degradation in the intestine with the consequence of alleviating insulin resistance and metabolic disorders aggravated by these metabolites.

## 5. Conclusions

In this study, the administration of SF-Alg remarkably decreased the levels of FBG, BW, food and water intake, and serum lipid in HFD/STZ-induced diabetic mice. Additionally, SF-Alg alleviated the histopathological injury and redox imbalance. SF-Alg remodeled diabetic gut microbiota, meanwhile, reduced the levels of BCAAs and AAAs in the colon, which may be the underlying mechanism of the beneficial effects of SF-Alg on the T2D mice. Taken together, SF-Alg could be considered as a potential functional food or adjuvant agent for T2D.

## Figures and Tables

**Table 1:** SF-Alg improves serum lipid levels in HFD/STZ-induced diabetic mice.

Groups	TG (mmol/L)	TC (mmol/L)	LDL-c (mmol/L)	HDL-c (mmol/L)
NC	5.241 ± 1.58 ***	2.862 ± 0.53 *	0.702 ± 0.31 ***	0.46 ± 0.22
DC	13.502 ± 1.52	5.462 ± 2.30	1.752 ± 0.63	0.45 ± 0.04
SF-Alg	11.706 ± 0.73 *	3.624 ± 0.59 *	1.53 ± 0.38	0.57 ± 0.11 *

Data are expressed as mean ± SD. * Indicates significantly different from the DC group at the * *p* < 0.05, *** *p* < 0.001 level. NC, normal control group; DC, diabetic control group; SF-Alg, diabetes + SF-Alg group; TG, triglycerides; HDL-c, high-density lipoprotein cholesterol; LDL-c, high-density lipoprotein cholesterol.

## Data Availability

The data that support the findings of this study are available from the corresponding author upon reasonable request.

## Supplementary Materials

The following are available online at https://www.mdpi.com/article/10.3390/nu13082887/s1. Figure S1. FT-IR spectra of SF-Alg; Figure S2. SF-Alg alters the gut microbiota structure. (A) PCoA score plot of gut microbiota at the genus level; (B) NMDS score plot of gut microbiota; Figure S3. SF-Alg improves the gut integrity in the diabetic mice. The plasma lipopolysaccharide (LPS); Figure S4. Spearman’s correlation between gut microbiota and obesity-related indexes (Genus). Good’s coverage and the genus with significant correlations are shown; Figure S5. Effect of SF-Alg on the colonic metabolite profile of the diabetic mice. Box plot visualizations of the relative abundances of BCAAs (L-valine and L-isoleucine) and AAAs (L-tyrosine and L-phenylalanine) metabolites in SF-Alg and DC groups; Table S1. Effect of SF-Alg on diversity of gut microbiota in the diabetic mice.

## Abbreviations

SF-Alg, *Sargassum fusiforme* alginate; T2D, type 2 diabetes; STZ, streptozotocin; HFD, high-fat diet; BW, body weight; FBG, fasting blood glucose; IPGTT, intraperitoneal glucose tolerance test; AUC, the area under the curve; TC, total cholesterol; TG, triglycerides; HDL-c, high-density lipoprotein cholesterol; LDL-c, high-density lipoprotein cholesterol; MDA, maleic dialdehyde; SOD, superoxide dismutase; CAT, catalase; BCAAs, branched-chain amino acids; AAAs, aromatic amino acids; LC-MS, liquid chromatography-mass spectrometry; OTUs, Operational Taxonomic Units; PCoA, Principal Co-ordinates Analysis; NMDS, Non-Metric Multi-Dimensional Scaling; UPGMA, Unweighted Pair-group Method; LEfSe, linear discriminant analysis effect size; PLS-DA, partial least squares-discriminant analysis; OPLS-DA, orthogonal partial least-squares discriminant analysis; VIP, variable importance in the projection.
