# Supplementary material for: Sargassum fusiforme Alginate Relieves Hyperglycemia and Modulates Intestinal Microbiota and Metabolites in Type 2 Diabetic Mice
Source: Nutrients. 2021 Aug 22;13(8):2887. doi: 10.3390/nu13082887 (PMC8398017; doi:10.3390/nu13082887)
Supplement: Supplementary file 1 [file nutrients-13-02887-s001.zip › nutrients-1343301-supplementary materials.pdf]

SUPPLEMENTARY METHODOLOGIES:

1. Methodologies of gut microbiota by 16S rRNA amplicon sequencing

Sample preparation: Total fecal DNA was extracted using CTAB/SDS method. The V3-V4 region of the 16S rRNA was amplified using the universal primers 341F and 806R. All PCR reactions were carried out with Phusion® High-Fidelity PCR Master Mix (New England Biolabs). Then, the mixture PCR products were purified with GeneJET Gel Extraction Kit (Thermo Scientific). Sequencing libraries were generated using Ion Plus Fragment Library Kit (Thermo Scientific) following manufacturer's recommendations. The library quality was assessed on the Qubit 2.0 Fluorometer (Thermo Scientific). At last, the library was sequenced on an Ion S5™ XL platform (Thermo Scientific) and 400 bp/600 bp single end reads were generated.

Data acquisition: Paired-end reads were merged using FLASH (V1.2.7), and the Raw fastq files were processed by QIIME (version 1.7.0). Sequence analysis was performed by Uparse software (v7.0.1001), and Sequences with ≥ 97% identity were assigned to the same Operational Taxonomic Units (OTUs).

Data analysis: Refraction analysis, Good's coverage and alpha diversities were performed using Mothur v.1.30.1. The community richness was evaluated by Chao and ACE. The community diversity was evaluated by Shannon and Simpson index. A heatmap (Figure 5A) based on the relative abundance of OTUs was generated using pheatmap package in R-3.1.0. Non-Metric Multidimensional Scaling (NMDS) was employed to visualize the relationships between samples by two-dimensional ordination plotting using the vegan package in R-3.1.0. Weighted and unweighted principal coordinate analyses (PCoA) were performed using Mothur (version 28 V.1.30.1).

2. Methodologies of non-targeted metabolome analysis

Metabolites extraction from colon contents (Ng, Ryan, Trengove, & Maker, 2012; Ponnusamy, Choi, Kim, Lee, & Lee, 2011)**:** The distal colon contents were added ddH_2O (4°C) and mixed. 100 mg of sample was extracted with 1000 μL of pre-cooled methanol (-20°C). After centrifugation, the supernatant was evaporated and finally dissolved in 400 μL methanol aqueous solution (1:1, 4°C). For the quality control (QC) samples, 20 μL of extract was taken from each sample and mixed. These QC samples were used to monitor deviations of the analytical results from these pool mixtures and compare them to the errors caused by the analytical instrument itself. And the rest of the samples were used for LC-MS detection.

UPLC Conditions (Sangster, Major, Plumb, Wilson, & Wilson, 2006)**:** Chromatographic separation was accomplished in an Acquity UPLC system equipped with an ACQUITY UPLC® HSS T3 (150 × 2.1 mm, 1.8 μm, Waters) column maintained at 4°C. The temperature of the auto sampler was 4°C. Gradient elution of analytes was carried out with 0.1% formic acid in water (A) and 0.1% formic acid in acetonitrile (B) at a flow rate of 0.25 mL/min. Injection of 5 μL of each sample was done after equilibration. An increasing linear gradient of solvent B (v/v) was used as follows: 0~1 min, 2% B; 1~9.5 min, 2%~50% B; 9.5~14 min, 50%~98% B; 14~15 min, 98% B; 15~15.5 min, 98%~2% B; 15.5~17 min, 2%.

Mass spectrometry conditions (Liu et al., 2017)**:** The ESI-MSn experiments were executed on the Thermo LTQ Orbitrap XL mass spectrometer with the spray voltage of 4.8 kV and -4.5 kV in positive and negative modes, respectively. Sheath gas and auxiliary gas were set at 45 and 15 arbitrary units, respectively. The capillary temperature was 325°C. The voltages of capillary and tube were 35 V and 50 V, -15 V and -50 V in positive and negative modes, respectively. The Orbitrap analyzer scanned over a mass range of m/z 89-1000 for full scan at a mass resolution of 60000. Data dependent acquisition (DDA)

MS/MS experiments were performed with CID scan. The normalized collision energy was 30 eV. Dynamic exclusion was implemented with a repeat count of 2, and exclusion duration of 15 s.

Data processing (Wang, Garrity, Tiedje, & Cole, 2007)**:** UPLC-QTOF-MS raw data were analyzed with MarkerLynx Application Manager 4.1 (Waters Corp.). The matrix from UPLC-QTOF-MS was introduced into SIMCA-P 11.0 software (Umetrics) and standardized to a mean of 0 and variance of 1, according to the formula [X - mean(X)]/ std (X), for multivariate statistical analysis. The t test with false discovery rate correction was used to measure the significance of each metabolite. Partial least-squared discriminant analysis (PLS-DA) and orthogonal partial least-squared discriminant analysis (OPLS-DA) were conducted to identify the metabolite discrimination between the two group samples. Differential metabolites were defined with variable importance in the projection (VIP) > 1.0 obtained from OPLS-DA and *P* values less than 0.05 obtained from t test. Differential metabolites were tentatively identified by database matching, i.e., Human Metabolome Database (HMDB) (http://www.hmdb.ca), Metlin (http://metlin.scripps.edu), massbank (http://www.massbank.jp/), LipidMaps (http://www.lipidmaps.org), mzclound (https://www.mzcloud.org). Heatmaps of differential metabolites between the SF-Alg-treated group and DC group were obtained based on spearman correlation and cluster analyses.

References

Liu, R., Hong, J., Xu, X., Feng, Q., Zhang, D., Gu, Y., et al. (2017). Gut microbiome and serum metabolome alterations in obesity and after weight-loss intervention. *Nature Medicine*, *23*, 859-868.

Ng, J. S. Y., Ryan, U., Trengove, R. D., & Maker, G. L. (2012). Development of an untargeted metabolomics method for the analysis of human faecal samples using Cryptosporidium-infected samples. *Molecular and Biochemical*

Parasitology, *185*, 145-150.

Ponnusamy, K., Choi, J. N., Kim, J., Lee, S.-Y., & Lee, C. H. (2011). Microbial community and metabolomic comparison of irritable bowel syndrome faeces. *Journal of Medical Microbiology*, *60*(6), 817-827.

Sangster, T., Major, H., Plumb, R., Wilson, A. J., & Wilson, I. D. (2006). A pragmatic and readily implemented quality control strategy for HPLC-MS and GC-MS-based metabonomic analysis. *Analyst*, *131*, 1075-1078.

Wang, Q., Garrity, G. M., Tiedje, J. M., & Cole, J. R. (2007). Naive Bayesian classifier for rapid assignment of rRNA sequences into the new bacterial taxonomy. *Applied and Environmental Microbiology*, *73*, 5261-5267.

Supplementary materials

Table S1. Effect of SF-Alg on diversity of gut microbiota in the diabetic mice.

	**NC**	**DC**	**SF-Alg**
Reads	80101±1384.81	81598±5256.35	80491±981.53
Observed species	461±73.79**	374±71.47	382±65.27
Shannon	5.24±0.59	4.82±0.65	4.90±0.72
Simpson	0.91±0.044	0.89±0.5	0.91±0.044
Chao1	582.79±1133.02**	433.43±80.04	455.84±81.40*
Ace	581.73±120.09**	438.63±75.99	455.54±75.87*
Goods coverage	0.99775±0.00046	0.998±0.00075	0.998±0.00053

Data are presented as mean ± SEM. * P<0.05, ** P < 0.01 compared to DC group.

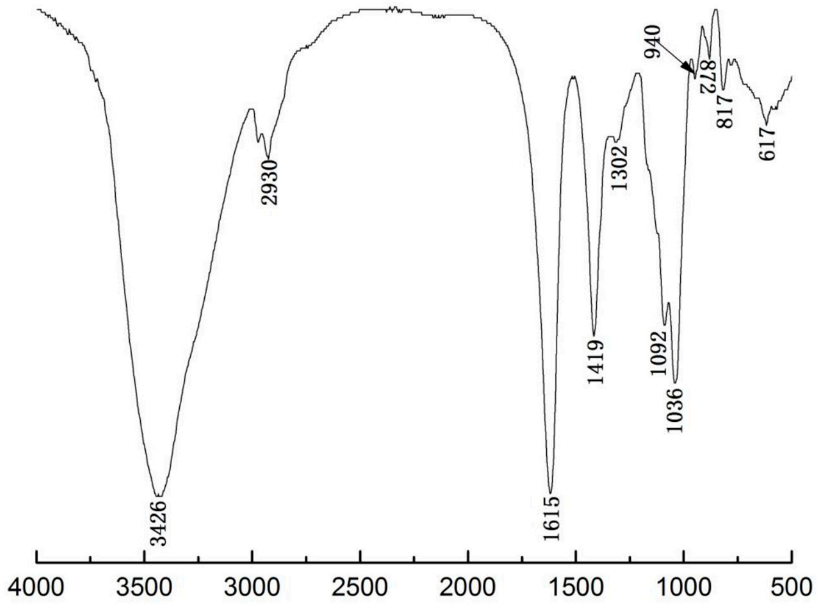

Figure S1. FT-IR spectra of SF-Alg.

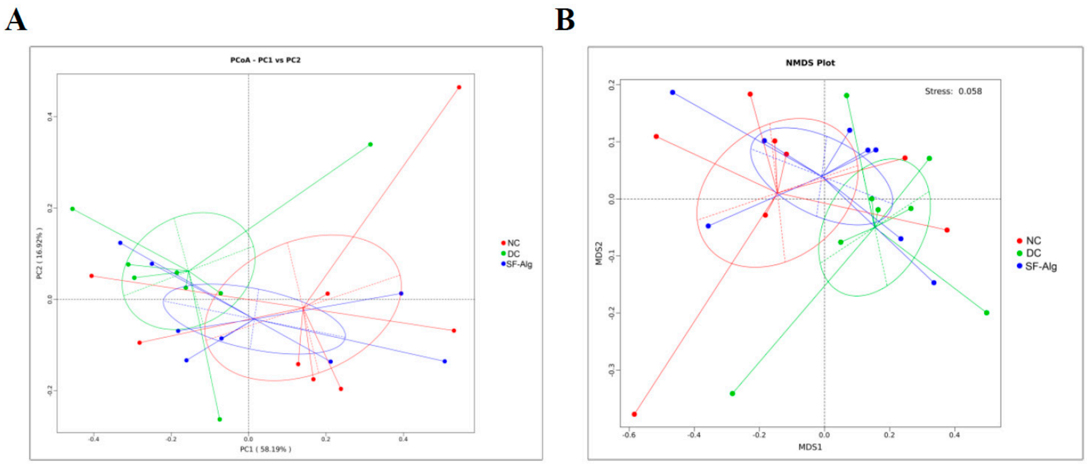

Figure S2. SF-Alg alters the gut microbiota structure. (A) PCoA score plot of gut microbiota at the genus level; (B) NMDS score plot of gut microbiota.

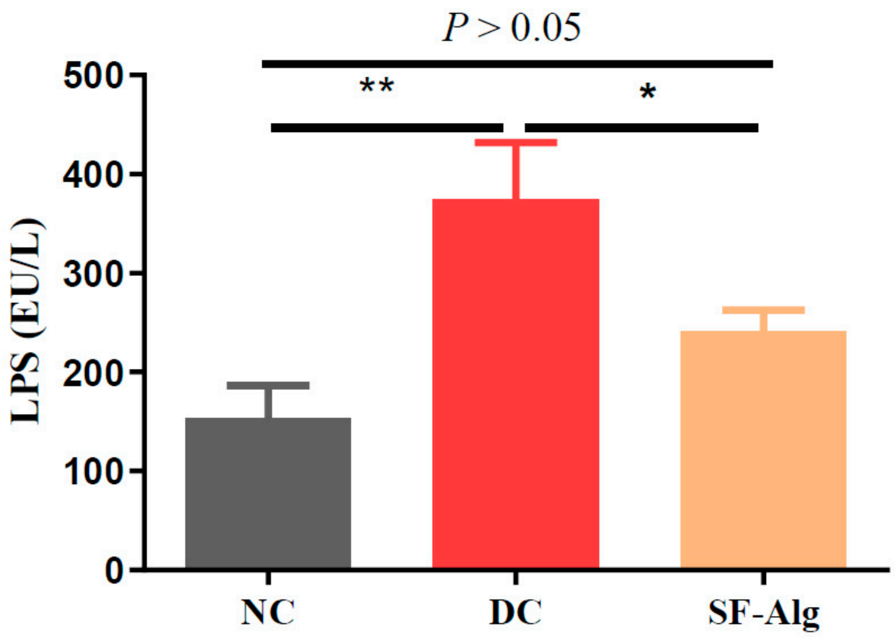

Figure S3. SF-Alg improves the gut integrity in the diabetic mice. The plasma lipopolysaccharide (LPS).

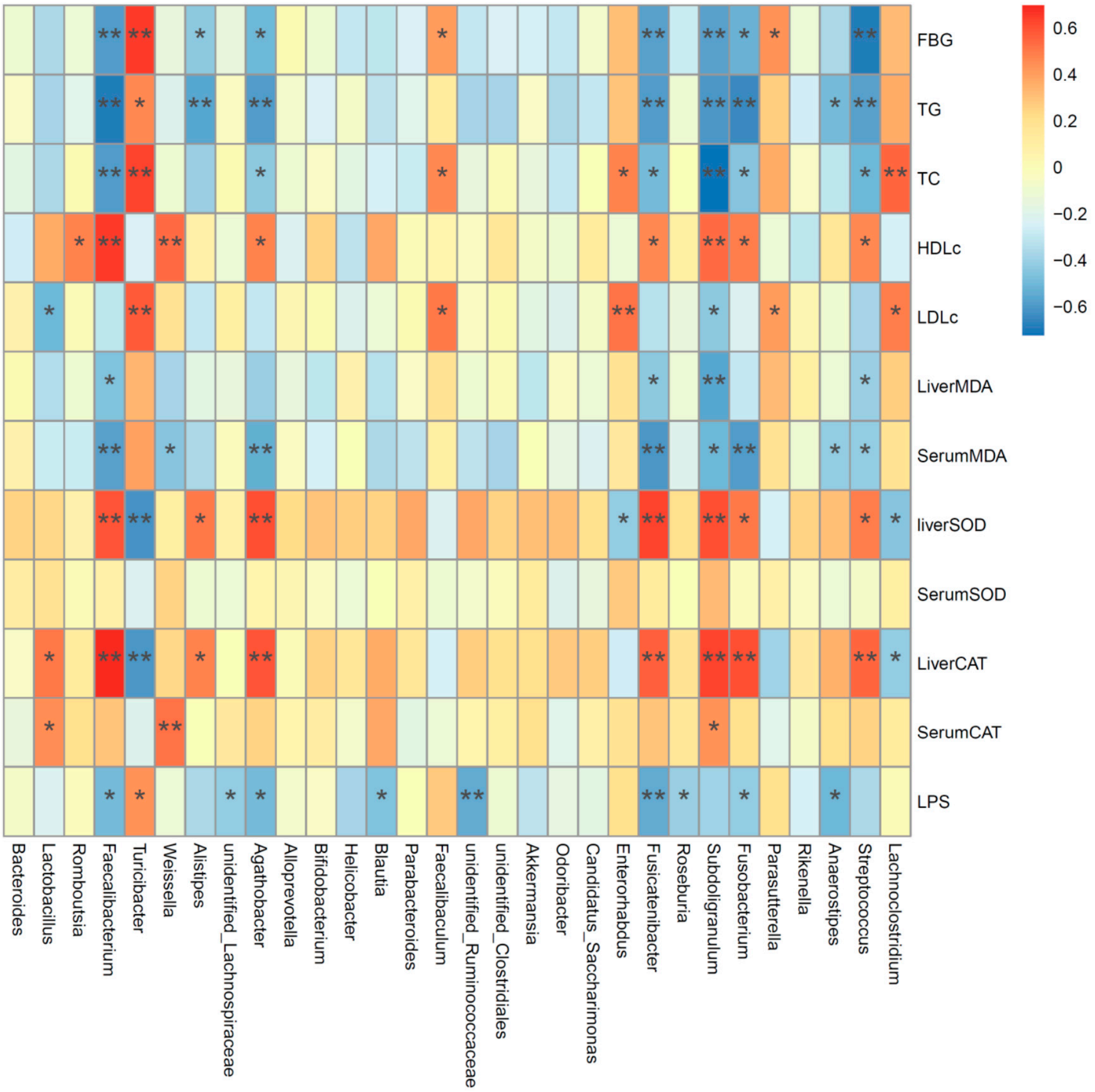

Figure S4. Spearman's correlation between gut microbiota and obesity-related indexes (Genus). Good's coverage and the genus with significant correlations are shown.

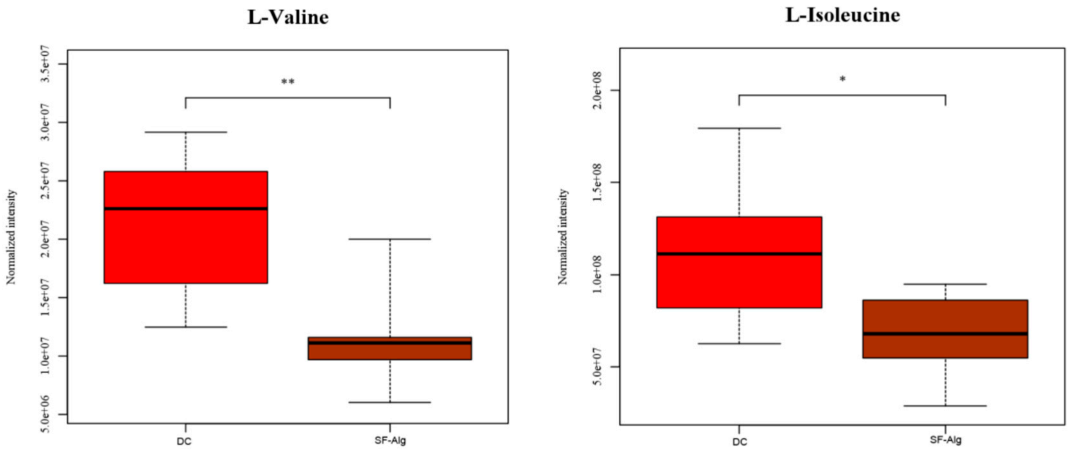

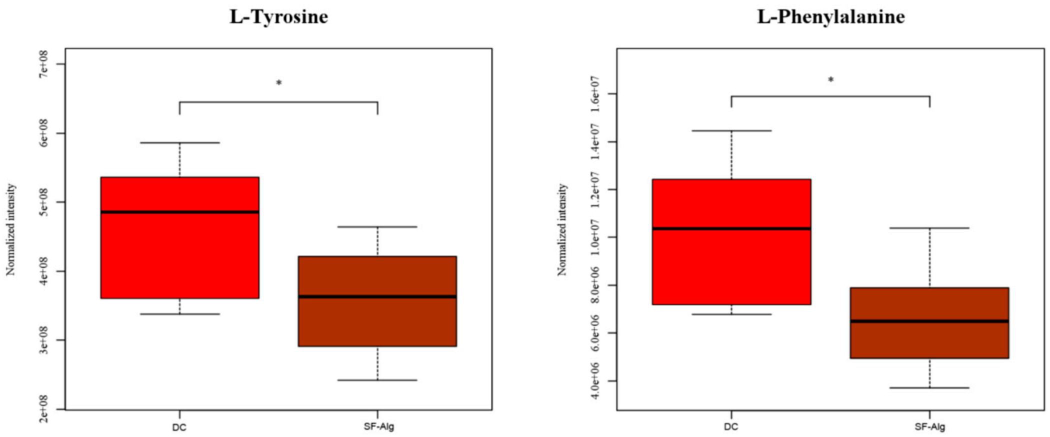

Figure S5. Effect of SF-Alg on the colonic metabolite profile of the diabetic mice. Box plot visualizations of the relative abundances of BCAAs (L-valine and L-isoleucine) and AAAs (L-tyrosine and L-phenylalanine) metabolites in SF-Alg and DC groups.
